# Docetaxel *vs* 5-fluorouracil plus vinorelbine in metastatic breast cancer after anthracycline therapy failure

**DOI:** 10.1038/sj.bjc.6600645

**Published:** 2002-11-12

**Authors:** J Bonneterre, H Roché, A Monnier, J P Guastalla, M Namer, P Fargeot, S Assadourian

**Affiliations:** Centre Oscar Lambret, 3 rue F. Combemale, 59020 Lille, France; Centre Claudius Regaud, Service Oncologie, 20–24 rue du Pont-Saint-Pierre, 31052 Toulouse, France; CHG André Boulloche, Service Oncologie, 2 rue du Dr Flamand, 25209 Montbelliard, France; Centre Léon Berard, 28 rue Laënnec, 69373 Lyon, France; Centre Antoine Lacassagne, 3 av de Valombrose, 06189 Nice, France; Centre Georges-François Leclerc, 1 rue du Pr. Marion, 21034 Dijon, France; Aventis Pharma, 20 Av Raymond Aron, Croix-de-Berny, 92165 Antony, France

**Keywords:** metastatic breast cancer, anthracycline, docetaxel, 5-fluorouracil, second-line chemotherapy, vinorelbine

## Abstract

This multicentre, randomised phase III study compared docetaxel with 5-fluorouracil+vinorelbine in patients with metastatic breast cancer after failure of neo/adjuvant or one line of palliative anthracycline-based chemotherapy. One hundred and seventy-six metastatic breast cancer patients were randomised to receive docetaxel (100 mg m^−2^) every 3 weeks or 5-fluorouracil+vinorelbine: 5-fluorouracil (750 mg m^−2^ per day continuous infusion) D1–5 plus vinorelbine (25 mg m^−2^) D1 and D5 of each 3-week cycle. Eighty-six patients received 516 cycles of docetaxel; 90 patients received 476 cycles of 5-fluorouracil+vinorelbine. Median time to progression (6.5 *vs* 5.1 months) and overall survival (16.0 *vs* 15.0 months) did not differ significantly between the docetaxel and 5-fluorouracil+vinorelbine arms, respectively. Six (7%) complete responses and 31 (36%) partial responses occurred with docetaxel (overall response rate 43%, 95% confidence interval: 32–53%), while 4 (4.4%) complete responses and 31 (34.4%) partial responses occurred with 5-fluorouracil+vinorelbine (overall response rate 38.8%, 95% confidence interval: 29–49%). Main grade 3–4 toxicities were (docetaxel *vs* 5-fluorouracil+vinorelbine): neutropenia 82% *vs* 67%; stomatitis 5% *vs* 40%; febrile neutropenia 13% *vs* 22%; and infection 2% *vs* 7%. There was one possible treatment-related death in the docetaxel arm and five with 5-fluorouracil+vinorelbine. In anthracycline-pretreated metastatic breast cancer patients, docetaxel showed comparable efficacy to 5-fluorouracil+vinorelbine, but was less toxic.

*British Journal of Cancer* (2002) **87**, 1210–1215. doi:10.1038/sj.bjc.6600645 www.bjcancer.com

© 2002 Cancer Research UK

## 

Metastatic breast cancer (MBC) is sensitive to chemotherapy but remains incurable with current therapeutic approaches. Single agents such as doxorubicin or epirubicin, cyclophosphamide, 5-fluorouracil (5-FU) and methotrexate achieve overall response rates (ORRs) ranging from 20 to 50% in this setting (Henderson *et al*, 1987; Clavel and Catimel, 1993). Combinations of alkylating agents with anthracyclines are extensively used in MBC and yield ORRs ranging from 40 to 60%, with complete response rates <20%, and a median response duration <15 months (The French Epirubicin Study Group, (1991); Brufman *et al*, 1997; del Mastro *et al*, 2001).

Use of anthracyclines is associated with problems of cumulative cardiotoxicity and primary or secondary resistance. As a result, there is a limit to the cumulative dose and number of regimens that can be administered to any given patient. Subsequent therapy in case of treatment failure is, therefore, a problem. Single-agent docetaxel has been proposed as an alternative treatment for patients previously treated with anthracycline-based therapy; the efficacy of this drug has been demonstrated in pretreated patients with MBC (Ravdin *et al*, 1995; Valero *et al*, 1995; Nabholtz and Crown, 1998; Alexopoulos *et al*, 1999; Brodowicz *et al*, 2000).

In patients with anthracycline-resistant MBC, docetaxel (100 mg m^−2^), infused over 1 h every 3 weeks, induces ORRs ranging from 30 to 69% (Ravdin *et al*, 1995; Valero *et al*, 1995; Nabholtz and Crown, 1998; Alexopoulos *et al*, 1999; Brodowicz *et al*, 2000).

Single-agent vinorelbine, in second-line or salvage chemotherapy for MBC, has a reported response rate of approximately 16% (Degardin *et al*, 1994; Gasparini *et al*, 1994), and the effectiveness of continuous infusion 5-FU in adenocarcinomas is well established (Caballero *et al*, 1985). A combination of vinorelbine plus 5-FU (FUN) has been administered to patients who have failed anthracycline therapy. In a phase II study, vinorelbine (30 mg m^−2^ D1) plus 5-FU (350 mg m^−2^ per day continuous infusion D1–3) in pretreated MBC patients exhibited substantial activity (ORR 43%) and acceptable tolerability, with the main toxic effect being severe neutropenia in 24% of patients (Froudarakis *et al*, 1998). In another phase II study, first-line administration of FUN (vinorelbine 30 mg m^−2^ D1 and D5 plus 5-FU 750 mg m^−2^ per day continuous infusion D1–5) resulted in an ORR of 62% (Dieras *et al*, 1996). Main grade 3–4 toxicities comprised neutropenia (90%), mucositis (37%) and infection (13%). Nevertheless, the majority of patients received treatment on an outpatient basis. Such results culminated in the use of the FUN regimen in advanced breast cancer in France.

In this randomised phase III trial, we compared the efficacy and safety of single-agent docetaxel *vs* FUN in patients with MBC who had relapsed after anthracycline-based chemotherapy comprising neoadjuvant or adjuvant treatment, or one line of palliative chemotherapy. The schedules used are based on the results of previous studies (Dieras *et al*, 1996; Froudarakis *et al*, 1998).

## MATERIALS AND METHODS

### Patient selection

Patients were eligible for the study if they had histologically confirmed MBC, had been pretreated with one anthracycline-based chemotherapy regimen, were female and were aged >18 years. All patients were required to have measurable or evaluable disease; a World Health Organisation (WHO) performance status (PS) ⩽2; a 4-week wash-out period after any antitumour treatment; and adequate haematological, liver and renal functions. Written informed consent was obtained from each patient before study treatment. The exclusion criteria were: only locally advanced disease; prior therapy with either taxanes or vinorelbine; more than one line of prior palliative chemotherapy; CNS involvement; osteoblastic bone lesions, carcinomatous lymphangitis of the lung or serous effusions as the only sites of disease; sensory neuropathy ⩾grade 2; or any severe concomitant condition, including coronary insufficiency.

As in previous docetaxel studies, patients were classified as anthracycline-sensitive or -resistant/refractory based on the following definitions: *refractory (*progressive disease during neoadjuvant or adjuvant chemotherapy, or progressive disease as the best response to palliative chemotherapy); *resistant* (relapse within the 12 months following either adjuvant or neoadjuvant chemotherapy, or disease progression on palliative chemotherapy after initial response); *potentially sensitive* (relapse more than 12 months after adjuvant chemotherapy or disease progression more than 4 weeks after the end of palliative chemotherapy) (Nabholtz and Crown, 1998).

### Drug administration

Patients were randomly assigned on a one-to-one basis to 1 of 2 groups, stratified by accruing centre. Patients received either docetaxel (Taxotere®, Aventis Pharmaceuticals, Paris, France) 100 mg m^−2^ over a 1-h i.v. infusion every 3 weeks, or 5-FU 750 mg m^−2^ per day continuous infusion on five consecutive days plus vinorelbine 25 mg m^−2^ over a 30-min infusion on days 1 and 5 of the 3-week cycle.

Premedication with an oral steroid (prednisolone 50 mg) was administered at 13, 7 and 1 h before each docetaxel infusion and then twice daily for the next 4 days. In both arms, treatment was planned for a maximum of nine cycles, except in the case of disease progression, unacceptable toxicity or consent withdrawal. Where significant toxicity occurred (WHO grade 3–4 non-haematological toxicity, febrile neutropenia or cycle delay >2 weeks) dose reductions were made in subsequent cycles. A maximum of two dose reductions per patient were allowed for both docetaxel (75 mg m^−2^, then 55 mg m^−2^) and 5-FU (600 mg m^−2^, then 500 mg m^−2^), and only one for vinorelbine (20 mg m^−2^). Dosing re-escalation was not allowed.

### Evaluation

Pretreatment evaluation consisted of a complete medical history and physical examination; complete blood cell count; biochemical profile and urinalysis; ECG; echocardiography or MUGA in case of known heart disease; measurement of all tumour-associated lesions by chest X-ray, abdominal ultrasound and/or computed tomography (CT) scan; and a bone scan complemented by X-ray, CT or magnetic resonance imaging of hot spots. Before each treatment cycle, patients had a physical examination, complete blood cell count, biochemical profile and urinalysis. A complete blood cell count was performed on day 5 of each cycle. Toxicity was evaluated according to WHO criteria (Miller *et al*, 1981). Antitumour activity was assessed every 3 cycles (after cycles 3, 6 and 9) on all target lesions. On day 28 after the last infusion, patients had a complete tumour evaluation, physical examination and ECG.

Tumour responses and time-related parameters were assessed according to WHO criteria (Miller *et al*, 1981). Time to progression (TTP) was calculated from the first treatment infusion to the first objective evidence of tumour progression. All responses documented by imaging were reviewed by an external panel of radiologists.

### Statistical methods

The primary endpoint of the study was TTP. In order to reject the null hypothesis of no difference between the two study arms, enrolment of 180 patients (90 per arm) was planned. The sample size provided the study with 85% power to detect a difference in progression free survival (after 9 cycles) of 60% in the docetaxel arm and 40% in the FUN arm, with a type I error of 0.05.

The Kaplan–Meier method was employed to analyse time-related parameters, and comparisons were performed using the non-parametric log rank test. The χ-test was used for non-censored qualitative parameters and Student's *t*-test for non-censored quantitative parameters. A multivariate analysis for prognostic factors was performed using a Cox proportional hazards model.

## RESULTS

### Patient characteristics

Between June 1995 and July 1997, 178 patients (docetaxel: 88; FUN: 90) were enrolled into the study in 22 centres. However, two patients in the docetaxel arm did not receive treatment – one due to brain metastases diagnosed after randomisation, and the other due to consent withdrawal – yielding 176 treated patients. (Although the planned total of 180 patients were unavailable for recruitment, the 176 treated patients were sufficient for achieving the statistical hypothesis.) Baseline characteristics of the 176 treated patients (Table 1

**Table 1:**
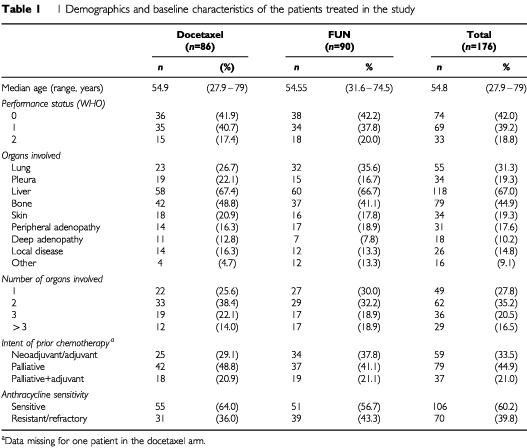
1 Demographics and baseline characteristics of the patients treated in the study

) were well balanced between the two treatment groups. The median age was 54.8 years, and 81% of patients had a PS⩽1. The median number of organs involved was two. The majority of patients (67%) had liver metastases.

All patients (except one in the docetaxel group, who was nonetheless considered as eligible without major protocol deviation) had received prior treatment with anthracycline-based chemotherapy; 66% of these patients received prior chemotherapy for advanced disease. According to the definitions for anthracycline sensitivity/resistance, 106 (60%) of the patients were potentially anthracycline-sensitive, including 22% relapsing more than 12 months after the completion of an adjuvant therapy.

### Treatment administration

Study treatment comprised 516 cycles of docetaxel (median per patient: 6; range: 1–12) and 476 cycles of FUN (median per patient: 6; range: 1–9). In the docetaxel arm, dose reductions were made in 75 of the 430 cycles in which a reduction was allowed (17%) and in the FUN arm, 171 of the 386 cycles were reduced (44%). Delays longer than 7 days occurred in 17 cycles in the docetaxel arm (3.9%) and 96 cycles in the FUN arm (25%). Consequently, the relative dose intensity of docetaxel was 0.97 while those of 5-FU and vinorelbine were 0.88 and 0.84, respectively.

### Efficacy

Results are presented for the intention-to-treat populations, unless otherwise indicated.

### Primary endpoint: time to progression

**Figure 1:**
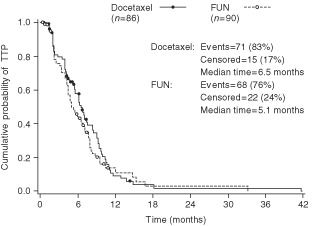
Time to tumour progression in the all-treated population.

**Figure 2:**
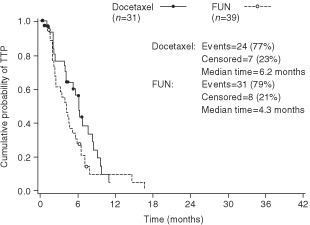
Time to tumour progression in the anthracycline-resistant/ refractory population.

As of 30 November 1998, the median follow up was 30.3 months (range 10.4–45.0 months) with 15 patients (17%) in the docetaxel arm and 22 (24%) in the FUN arm having experienced no disease progression at the cut-off date. The median TTP was 6.5 months (95% CI: 5.5–8.4 months) in the docetaxel arm (15 patients censored) and 5.1 months (95% CI: 4.4–6.9 months) in the FUN arm (22 patients censored; *P*=0.34; Figure 1

). When only the 70 anthracycline-resistant/refractory patients were taken into account, the median TTP was 6.2 months in the docetaxel arm (seven patients censored) and 4.3 months in the FUN arm (eight patients censored; *P*=0.13; Figure 2

).

### Secondary endpoints

In the docetaxel arm there were six complete responses (CRs) (7%) and 31 partial responses (PRs) (36%), giving an ORR of 43%. In the FUN arm there were four CRs (4.4%) and 31 PRs (34.4%), giving an ORR of 39% (see Table 2

**Table 2:**
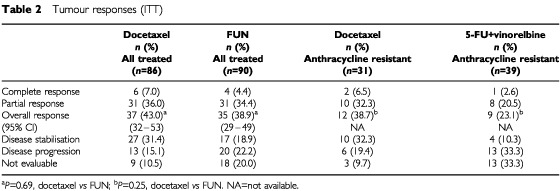
Tumour responses (ITT)

). The difference in ORR was not statistically significant (*P*=0.69). The median duration of objective responses was 8.4 months in the docetaxel arm and 7.8 months in the FUN arm. The ORRs in evaluable patients with liver, bone or lung metastases were 38, 51 and 53%, respectively, in the docetaxel arm; and 49, 44 and 52%, respectively, in the FUN arm.

The ORRs in evaluable patients with 1, 2, 3 or >3 organs involved, were 43, 52, 53 and 40%, respectively, in the docetaxel arm; and 57, 48, 31 and 53%, respectively, in the FUN arm. These rates did not differ significantly between the two arms.

**Figure 3:**
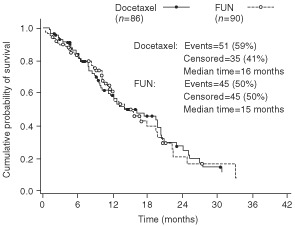
Overall survival in the all-treated population.

There was no difference between the two arms in overall survival (OS). The median OS was 16 months for docetaxel (35 patients censored) and 15 months for FUN (45 patients censored) (Figure 3

).

**Figure 4:**
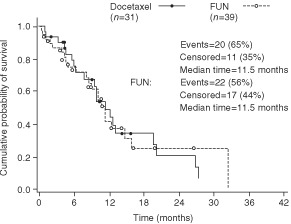
Overall survival in the anthracycline-resistant/refractory population.

When only the 70 anthracycline-resistant/refractory patients were taken into account, docetaxel yielded an ORR of 39% (12/31 patients) *vs* 23% (9/39 patients) for FUN, and the median survival was 11.5 months in both the docetaxel and FUN arms (Figure 4

).

### Univariate and multivariate analysis

The objective of these analyses was to determine significant prognostic factors for TTP. First, a univariate analysis was performed on 12 factors, and all factors found to have a statistical significance (at the level of *P*<0.20) were carried forward for a multivariate analysis using a Cox proportional hazards model. Of the 12 prognostic factors included in the univariate analysis, four were found to correlate with TTP. The multivariate stepwise analysis confirmed that two of these four factors (anthracycline sensitivity and the number of organs involved) were significantly correlated with the TTP (Table 3

**Table 3:**
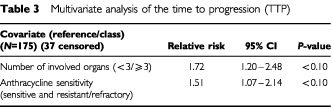
Multivariate analysis of the time to progression (TTP)

). A patient having ⩾ three organs involved had a 1.72 greater risk of progression than a patient with two or fewer involved organs, and prior anthracycline resistance increased the risk of progression by 1.51.

### Safety

The safety profiles for each treatment arm are summarised in Table 4

**Table 4:**
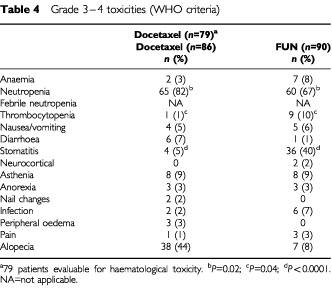
Grade 3–4 toxicities (WHO criteria)

. The main toxicity (WHO grade 3–4) in both treatment arms was neutropenia. Grade 3–4 neutropenia was significantly more frequent with docetaxel than with FUN (82 *vs* 67%, respectively; *P*=0.02), while severe thrombocytopenia and severe stomatitis were significantly more frequent with FUN than with docetaxel (10 *vs* 1%, respectively; *P*=0.02 and 40 *vs* 5%, respectively; *P*<0.0001). Febrile neutropenia occurred more frequently with FUN than with docetaxel (22 *vs* 13%, respectively; *P*=0.10), as did infection (grade 3–4) (7 *vs* 2%, respectively; *P*=0.28). Docetaxel led to more alopecia (67 *vs* 24%; *P*<0.0001) and sensory neuropathy (grade 1–2) (35 *vs* 6%; *P*<0.0001) than FUN. Characteristic cumulative severe dose-related side effects of docetaxel (i.e. fluid retention (peripheral oedema), nail dystrophy and skin reactions) were graded as severe in only 3, 2 and 0% of patients, respectively.

In the docetaxel arm, three patients died during the study: two from progressive disease, which was not considered to be related to treatment, and one from congestive heart failure, possibly related to treatment. In the FUN arm, nine patients died during the study. Five deaths were considered to be probably related to study treatment (three sepsis, one liver failure and one grade 4 diarrhoea and mucositis associated with liver and renal failure). Four deaths were not considered to be related to treatment: two died from disease progression, one from acute pulmonary oedema, one from hepato-cellular insufficiency.

## DISCUSSION

Prior to widespread taxane use, several second-line chemotherapy regimens were used after the failure of anthracycline-based regimens. These included vinorelbine plus mitomycin C (Vici *et al*, 1996), vinorelbine combined with cisplatin (Ray-Coquard *et al*, 1998), and 5-FU as single agent or combined with vinorelbine (Cany *et al*, 1996; Dieras *et al*, 1996; Froudarakis *et al*, 1998). The approval of docetaxel as a single agent in this setting, based on high response rates in phase II studies (Ravdin *et al*, 1995; Valero *et al*, 1995; Marty *et al*, 1997), prompted this randomised trial in which the efficacy and safety of docetaxel was compared with that of the FUN regimen, a popular treatment for advanced breast cancer patients in France.

The patient characteristics in this trial were representative of those for the general population of patients with MBC. Interestingly, 60% of the patients in this study were considered to be potentially sensitive to anthracycline according to available operational definitions. There was no evidence of any imbalance in baseline characteristics between the two groups.

In terms of efficacy, there were no significant differences in TTP, response rate, response duration or OS between the two treatments. There was, however, a trend towards both a higher ORR, particularly in anthracycline-resistant tumours, and a longer TTP with docetaxel. The response rate in anthracycline-resistant/refractory patients was 39% with docetaxel *vs* 23% with FUN. The small sample size of this sub-group (31 for docetaxel, 39 for FUN), does not allow pertinent statistical analysis of the two treatments.

The ORRs observed in both arms of the study are similar to those seen in other studies. The ORR of 43% for docetaxel reported in our study is similar to the ORR of 48% observed by Chan in a phase III study of MBC patients who had failed alkylating agent therapy (Chan *et al*, 1999). The ORR of 39% observed for the FUN regimen in the current study is similar to the 43% ORR reported by Froudarakis *et al* (1998) in patients with MBC who had all been pretreated, mainly with anthracyclines.

Interestingly, the ORR of 43% for docetaxel (for all patients pretreated with anthracyclines) observed in this study is better than the ORR of 30% observed by Nabholtz *et al* (1999), who compared docetaxel as a single agent against vinblastine and mitomycin C (VMC) in patients who had failed anthracycline therapy. However, patients who relapsed more than 12 months after adjuvant chemotherapy were not accepted, and 43% of patients considered not to be resistant had progressive disease more than 30 days after the completion of palliative chemotherapy. VMC was found to be better tolerated than docetaxel, but docetaxel led to a significantly improved response rate. Median TTP was significantly longer in the docetaxel arm than in the VMC arm (19 weeks *vs* 11 weeks; *P*=0.001), as was median OS (11.4 months *vs* 8.7 months in all randomised patients; *P*=0.01). The cohort enrolled in the Nabholtz study had a higher rate of anthracycline resistance and a poorer prognostic profile than the patients in our study, as is supported by their shorter TTP and survival. The Nabholtz study also enrolled 392 patients, including 220 (56%) true anthracycline-resistant patients, as opposed to 70 (40%) anthracycline-resistant patients in our study.

The safety profiles of the two treatments differed. Docetaxel induced higher incidence of alopecia, grade 1–2 peripheral neuropathy, fluid retention, skin and nail disorders and grade 3–4 neutropenia, whereas FUN was responsible for more grade 3–4 stomatitis and thrombocytopenia. Despite the lower incidence of severe neutropenia with FUN, patients receiving this therapy experienced more febrile neutropenia and neutropenic infections than patients receiving docetaxel. Sepsis was responsible for three deaths in the FUN group, whereas no patients died from a neutropenia-related complication in the docetaxel group. This striking mortality difference is probably due to the association of febrile neutropenia and stomatitis with the FUN regimen.

The feasibility of the FUN regimen in this trial was compromised by poor compliance. There were more frequent dose reductions and dose delays, which were responsible for lower relative dose intensities of 5-FU and vinorelbine than docetaxel. The FUN regimen chosen was based on the results of studies carried out before this trial was started (Dieras *et al*, 1996; Froudarakis *et al*, 1998). However, more recent trials indicate that other FUN regimens may be less toxic (Lombardi *et al*, 2000; Zambetti *et al*, 2000).

In conclusion, single-agent docetaxel (100 mg m^−2^) is as effective as, and less haematologically toxic than, the chosen FUN regimen. Furthermore, the single 1-h docetaxel infusion is more convenient than the 5-day FUN regimen. Our results, together with those of another controlled trial (Nabholtz *et al*, 1999), demonstrate that docetaxel as a single agent is an important and easy-to-use treatment option in MBC after anthracycline failure.
